# Diverse roles of quaking in endothelial cell biology

**DOI:** 10.1007/s10456-025-10020-w

**Published:** 2025-11-12

**Authors:** Lincy Edatt, Danyan Li, Andrew C. Dudley, Chad V. Pecot

**Affiliations:** 1 UNC Lineberger Comprehensive Cancer Center, 450 West Drive, Chapel Hill, NC 27599 USA; 2 RNA Discovery Center, University of North Carolina, Chapel Hill, USA; 3 Department of Microbiology, Immunology, and Cancer Biology, University of Virginia, Charlottesville, VA USA; 4 UVA Comprehensive Cancer Center, University of Virginia, Charlottesville, USA; 5 Division of Hematology/Oncology, University of North Carolina, Chapel Hill, USA

**Keywords:** Quaking, Endothelial cells, Angiogenesis, RNA-binding protein

## Abstract

Quaking (QKI), a member of the signal transduction and activators of RNA (STAR) family of RNA-binding proteins, affects a wide range of functions, including alternative splicing, mRNA precursor processing, mRNA transport and localization, mRNA stabilization, and translation. Recently, QKI has been found to have critical roles in vasculogenesis and angiogenesis due to its effects on alternate splicing and other post-transcriptional modifications involving small RNAs in the endothelial cells (ECs). Aberrant expression or mutation of QKI in ECs can result in pro- or anti-angiogenic effects under different physiological and pathological conditions, including tumor angiogenesis. However, the regulatory roles of QKI in EC biology remain poorly described. This review summarizes our current understanding of the QKI isoforms and their functions in ECs, as well as the potential utility of QKI as an emerging translational target for angiogenic-based therapies.

## Introduction

RNA-binding proteins (RBPs) bind to specific RNA sequence motifs to form ribonucleoprotein complexes to regulate critical post-transcriptional processes. In addition to influencing RNA metabolism, RBPs regulate various cellular processes, including proliferation, differentiation, and apoptosis, across multiple cell types [1]. Quaking (QKI) is an RBP belonging to the STAR protein family, which stands for signal transduction and activators of RNA [2, 3]. QKI and other family members, SAM68, SF1, SLM-1, SLM-2, GLD-1, KEP1, Sam50 and Artemia Salina GRP33 make up the STAR pathway [4]. Prediction based on the phenotype of Quaking viable mice (QK^v^) with a deleted QKI gene regulatory regions shows that QKI can effectively regulate cell growth and differentiation [2, 5]. STAR proteins have been postulated to have multiple functions in developmental processes, including regulation of RNA homeostasis in response to extracellular and/or developmental signals [6]. Consistent with developmental roles, QKI is involved in embryonic vasculogenesis, visceral endoderm, and barrier function related to angiogenesis, as well as epithelial cell adhesion [7–9]. Furthermore, QKI regulates alternative splicing targets in epithelial and vascular cells to adjust complex cellular responses like cell differentiation in diseases [7, 10, 11]. QKI is also involved in epithelial–mesenchymal transition (EMT) in the tumor development of multiple epithelial-derived cancer types [12, 13]. Here, we review the current literature describing the wide range of RNA regulatory functions carried out by QKI to direct endothelial cell (EC) biology under normal physiologic and various pathologic conditions.

## RNA-binding protein roles of QKI isoforms

The *qk* gene produces at least five isoforms of QKI protein from six transcripts due to alternative splicing [5, 11] [14]. The major QKI isoforms, QKI-5, QKI-6, and QKI-7, are predicted to contain a QUA1 domain, a QUA2 domain, and a single KH RNA-binding domain [3]. QUA1 in GSG/STAR (GRP33, Sam68, GLD-1) mediates QKI self-association, and the absence of QKI self-association leads to embryonic lethality in QK^v^ mice, which carries a spontaneous, recessive mutation affecting the *Qki* gene locus [15, 16]. KH domain, a part of the conserved GSG protein domain (GRP33, Sam68, GLD-1), is involved in the regulation of cellular RNA metabolism, from pre-mRNA processing to translational regulation [17, 18]. For example, the overexpression of the KH domain-containing RBP, hnRNPK, increases breast cancer cell proliferation and growth through enhancing promoter activity and translation of oncogenes like c-Myc [19, 20]. The KH domain is not only involved in RNA–protein interaction with 3D βααββα folds, but also protein–protein interactions [18, 21]. KH domains are characterized by a single conserved KH motif, VIGXXGXXI, which binds a well-defined consensus RNA motif, ACUAA(C), and is required for the normal development of invertebrates [22]. Deletion of the KH motif substantially eliminates RNA-binding activity [22, 23]. Another domain, QUA2, flanks KH along with QUA1 on the other side, together forming the STAR/GSG domain, which is required for RNA binding and dimerization [24].

As a general rule, mRNA transcripts that share similar 3’UTR also show similarities in developmental expression [25]. However, the isoforms vary at their 3’ UTR, suggesting they may have different roles during development. For example, the selective loss of QKI-5 expression in severely disrupted oligodendrocytes suggests that the isoform specifically contributes to regulating early stages of myelination [5]. QKI-5 protein is also more abundant than the other QKI isoforms in early embryogenesis of the mouse, adding to its prominence in early myelination and neural plate formation [26]. After QKI-5’s expression peaks at day 0 in the nucleus, it gradually declines through embryogenesis, while QKI-6 and QKI-7 mRNA levels peak around 2 weeks in the cytoplasm after birth, approximately when myelination is completed [27, 28]. QKI-6 and 7 isoform subcellular localization corresponds to the function of 3’ UTR and intron binding rather than exon binding to control mRNA stability and splicing [5, 29]. Although both QKI-5 and QKI-6 mRNA contain QK binding sites in their 3’ UTR, only the high density of binding sites in QKI-5 allows this specific isoform to autoregulate its own protein expression. A low level of QKI-5 expression promotes its own mRNA accumulation through stabilization; however, high levels of QKI-5 result in translational inhibition by binding to the 3’UTR.

QKI-6 shares a similar mechanism of limiting protein expression, negatively impacting both itself and QKI-5 [10]. The isoforms also play the role of a potential inducer of apoptosis, regulating cancer development, and the shuttling ability of QKI-5 between cytoplasm and nucleus allows the three isoforms to function as mediators for nucleo-cytoplasmic translocation during signal transduction [9, 29]. QKI-5 is mostly restricted to the nucleus, whereas QKI-6 and QKI-7 are concentrated in the perikaryal cytoplasm (Table 1) [31]. The distinct localization of these isoforms is dictated by each isoform’s C termini, produced through alternative splicing events. The highly basic C terminus of QKI-5 acts as a nuclear localization signal [32]. Moreover, QKI actively regulates alternative splicing in a reciprocal manner. For example, QKI-6 determines the induction of VSMC differentiation through modulation of HDAC7 splicing [33]. Because most vertebrate QKI genes consist of two 3’ UTRs with distinct lengths, and their high levels of conservation across species, make it an ideal target for post-translational regulation by microRNAs (miRNAs) [13, 34, 35] For example, the miR-200 family has been found to directly target and suppress QKI-5, resulting in alternative splicing and inhibition of epithelial-mesenchymal transition [13]. Similarly, we previously demonstrated that miR-200 family inhibits tumor angiogenesis through inhibition of a QKI-CCND1 regulatory axis, which results in reduced EC proliferation [36]. QKI-5 was identified as a functional target of miR-221 by in silico methods. Overexpression of miR-221 reduced QKI-5 protein levels in human colorectal cancer (CRC) cells, whereas experiments showed that QKI-5 suppresses the tumor-forming capacity of human CRC cells [37]. Interestingly, QKI-7 impairs Hippo signaling pathways to sensitize cancer cells to chemotherapy. This is achieved through attenuating the translational efficiency of genes involved in the Hippo pathway. Under stress conditions, QKI7 interacts with the stress granule core protein G3BP1 and shuttles internal m^7^G-modified transcripts into stress granules, which results in regulation of mRNA stability and translation [38]. Additionally, QKI is reported to be closely associated with the protein encoded by the Colorectal Adenocarcinoma Hypermethylated gene; thus, QKI is expected to have a role in hypermethylation and other post-transcriptional regulations involving miRNAs [39].

**Table 1:** QKI Isoform localizations and functions

Isoform	Location	Function
QKI-5	Predominantly nuclear. Shuttles between the nucleus and cytoplasm in a transcription-dependent manner	Regulates alternative splicing of pre-mRNAs by binding within introns to promote either exon inclusion or skipping. It is a potent regulator of cellular differentiation, including myelination and epithelial-mesenchymal transition (EMT)
QKI-6	Primarily cytoplasmic, but can partially localize to the nucleus, especially when expressed at higher levels or when dimerized with QKI-5. Localizes to stress granules under cellular stress	Regulates mRNA stability and transport in the cytoplasm by binding to mRNA 3’ UTRs. Interacts with the cytoskeleton (microtubules and actin filaments) to direct mRNA localization. Co-localizes with Ago2 and PABP1 in stress granules
QKI-7	Primarily cytoplasmic, though it can enter the nucleus by forming a heterodimer with QKI-5. Also localizes to stress granules during cellular stress	Regulates mRNA stability and translation. Promotes the stability of certain microRNAs by recruiting cytoplasmic poly(A) polymerases to extend their poly(A) tails. It can bind to and traffic mRNAs along microtubules

## Post-translational modifications of QKI

Acetylation of STAR proteins upregulates their activity in human mammary epithelial cell lines, most significantly in tumorigenic cell lines [41]. The STAR proteins function as symmetric homodimers through alignments of helix-turn-helix folds and selectively bind to bipartite RNA sequences, forming stable hydrophobic interactions [42]. Dimer stability and the optimal RNA binding interfaces formed between QUA1, KH, and QUA2 domains increase QKI’s RNA binding affinity to approximately 100 nM, supporting multiple developmental functions including apoptosis and cell cycle regulation [3]. The protein can also determine an alternative splicing site by assembling with other splicing factors on introns [43].

All major QKI isoforms contain a couple of proline-rich putative Src-homology 3 (SH3) binding motifs at the C-terminus after the KH domain. SH3 mutation nullifies 70% tyrosine phosphorylation of QKI in the myelin compartment [44, 45]. Considering that Src-PTKs (e.g., p59fyn) can switch roles of RNA-binding proteins, such as changing Sam68 from a pro-apoptotic to an anti-apoptotic regulator in live cells, QKI may similarly regulate mRNAs via interaction with SH3-binding motifs [2, 46]. Mutants lacking the KH-domain of QKI-5 have QKI-6 and QKI-7 suppressed expression, reinforcing the significance of post-translational modification of QKI in isoform expression and activity [45, 47].

## Physiologic and pathologic QKI expression in micro-vessels

**Fig. 1:**
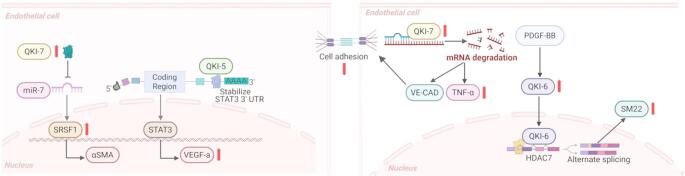
Molecular regulation of endothelial cell physiology and pathology by QKI

All QKI isoforms are expressed in macro-vascular and micro-vascular ECs of healthy arteries, most abundantly of which is QKI-5[48]. Most QKI isoforms are expressed in micro-vascular beds, peritubular capillaries, some glomerular endothelium, as well as kidney endothelium [49]. Vascular protective hemodynamic conditions, specifically against the laminar flow stress, have been found to induce QKI expression, with the greatest impact on the QKI-7 isoform [34]. Reduced QKI activity undermines the translation and expression of VE-Cadherin, as 3’UTRs in CDH5 mRNA present high-affinity quaking response elements (QREs) and results in impaired endothelial barrier function, causing a 40% increase in vascular leakage (Fig. 1) [48]. In ischemic microvessels, QKI-5 acts as a regulator of CD144 stabilization and VEGFR2 activation through STAT3 signaling during EC differentiation from iPSCs [50]. We have previously observed evidence that the tumor microvasculature has higher expression of all isoforms of QKI in both clinical and experimental lung cancer samples compared with matched normal lung endothelium. Furthermore, increased QKI expression in tumor endothelial cells (TECs) was associated with significantly worse overall survival in lung cancer patients, suggesting QKI may be a clinically relevant therapeutic target to regulate tumor angiogenesis [36].

## Roles of QKI in embryonic vasculogenesis

QKI regulates retinoic acid (RA) synthesis via Raldh2, which targets endothelial cell proliferation. This directly affects mesodermal-derived yolk sac vasculogenesis and indirectly EC migration [51, 52]. Since RA did not rescue vascular patterning nor increase the survivability of visceral endoderm apoptotic mutants, QKI, in addition, could have a direct target in visceral endoderm during embryonic vessel development [7]. Losing the QKI-5 splice site leads to poor endothelial cell (ECs) development and VSMC recruitment, causing failure in remodeling of yolk sac capillary plexuses, resulting in lethality of mouse embryos [48, 50]. QKI is highly expressed in yolk sac endoderm, the site of developing blood islands where differentiation of blood and endothelial cells occurs first. In addition, embryos homozygous for the *qk*^*k2*^ allele (T-to-A transversion at a specific nucleotide 977) have defective yolk sac vascular remodeling and abnormal vessels in the embryo at mid gestation, resulting in embryonic death [33, 53]. QKI also regulates smooth muscle cell differentiation during embryonic blood vessel formation. Investigation of QKI null embryos revealed that the vitelline artery was too thin to connect properly to the yolk sac. This prevented remodeling of the yolk sac vasculature, as the vitelline vessel was deficient in smooth muscle cells [54]. By binding to the splicing transcriptional co-activator, Myocad, QKI represses vascular smooth muscle cells’ (VSMC) ability to receive and stimulate responses to extracellular and intracellular signals. This pathway guides VSMC de-differentiation in response to vascular injury [55]. Decreased QKI expression inhibits VSMC proliferation, migration, and collagen production [7]. The inability of mutant mice to differentiate VSMCs leads to lethal instability and a lack of nascent vessels in the yolk sac vasculature [56].

## Roles of QKI isoforms in EC proliferation and differentiation

The QKI isoforms can have distinct functional roles in EC biology. For instance, induction of QKI‐5 during EC differentiation results in 3’UTR binding and stabilization of STAT3 expression, resulting in VEGFR transcriptional activation and VEGF secretion. Using iPS‐ECs overexpressing QKI, full recovery of blood flow was achieved in experimental hind limb ischemic models through improved angiogenesis and neovascularization [50]. In contrast, QKI-7 has been found to be highly expressed in human coronary arterial ECs of diabetic donors, and in blood vessels from diabetic critical limb ischemia patients who underwent lower-limb amputation. RNA splicing factors like CUG-BP and hnRNPM efficiently regulate QKI-7 expression through direct binding to QKI-7. QKI-7 upregulation correlated with disrupted cell barrier, compromised angiogenesis and enhanced monocyte adhesion. RNA immunoprecipitation (RIP) and mRNA-decay assays demonstrate that QKI-7 binds and promotes mRNA degradation of downstream targets CD144, Neuroligin 1 (NLGN1), and TNF-α-stimulated gene/protein 6 (TSG-6). When hindlimb ischemia is induced in diabetic mice and QKI-7 is knocked down in vivo in ECs, reperfusion and blood flow recovery are markedly promoted [49]. Thus, different isoforms of QKI compete to bind to VE-cadherin 3’UTR, resulting in distinct effects in EC barrier function and angiogenesis [5, 49].

During vascular injury, vascular smooth muscle cells (VSMCs) of the artery adopt a proliferative and contractile phenotype. QKI regulates VSMC phenotype by modulating the expression and alternative splicing of the myocardin pre-mRNA. Thus, QKI determines VSMCs’ ability in vessel repair and supports vascular tone after an injury. QKI-5 induced the splicing factor SF3B1 during EC differentiation in a time-dependent manner, implying that QKI-5 contributed to EC induction from iPSCs as an important splicing regulator [57]. QKI, as a UTR binding factor, could determine tissue-specific endothelial cell differentiation and lineage commitment, as it can regulate polyadenylation, splicing, and UTR regulatory factors to ensure tissue-specific protein isoforms in conjunction with canonical tissue-specific splicing factors like FOX-1/FOX-2 and CELF [49] [58].

## QKI in EC pathology

**Fig. 2:**
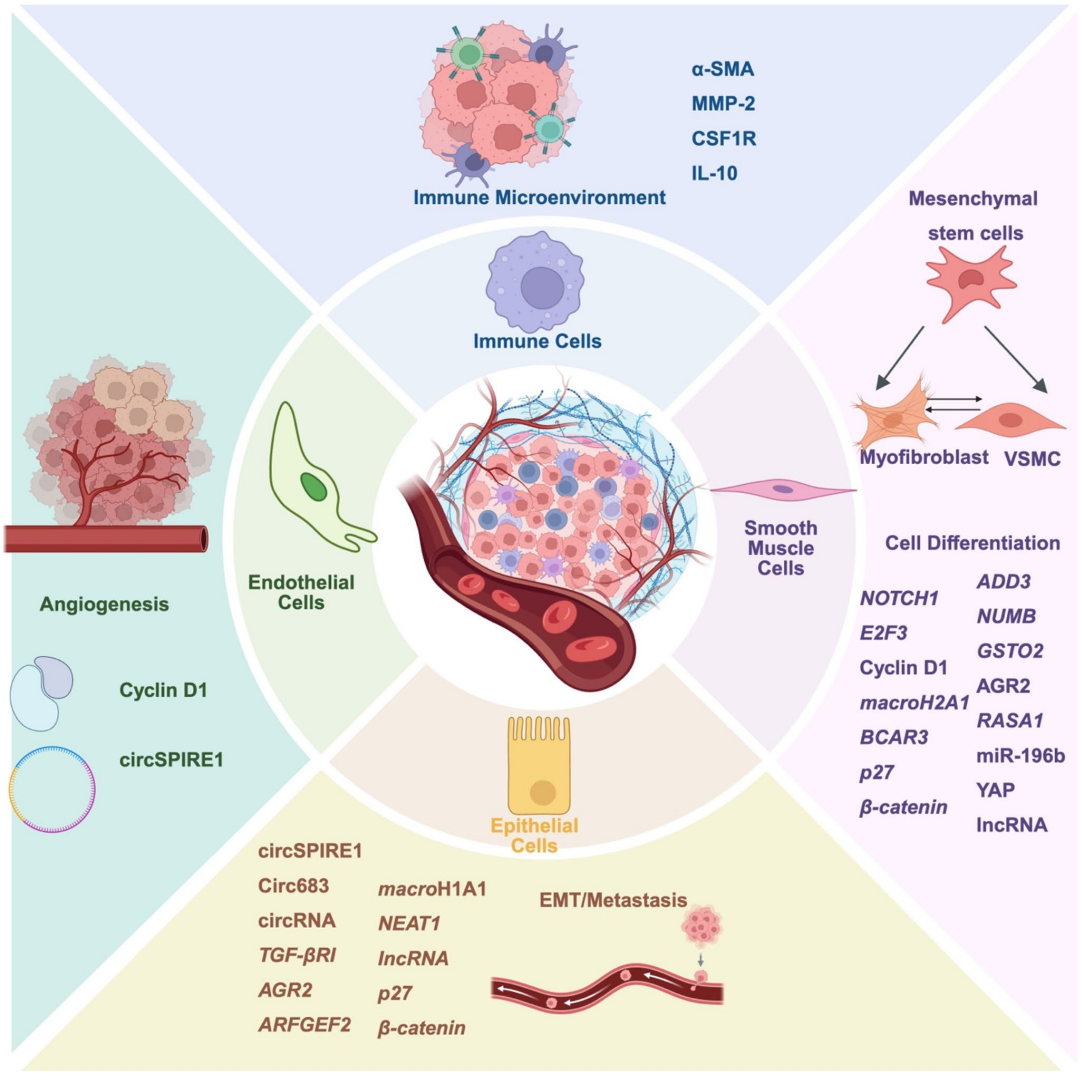
Direct and indirect roles of QKI in gene regulation on EC biology and functions

There are several diverse roles of QKI in endothelial pathophysiology depending on the underlying disorder (Fig. 2). Pulmonary arterial hypertension, characterized by elevation of pulmonary arterial pressure and vascular resistance, is often caused by extracellular matrix stiffness and leads to right ventricular remodeling and often death. Upregulation of QKI during PAH results in decreased levels of miR-7, causing de-repression and higher expression of the miR-7 target gene, serine and arginine-rich splicing factor 1 (SRSF1). This QKI-miR-7-SRSF1 axis contributes to increased ECM stiffness during PAH [59]. Dysfunction of ECs also has a key role in diabetic complications. Significant upregulation of QKI-7 has been observed in human iPS-ECs from diabetic patients when exposed to hyperglycemia [60]. QKI-7 overexpression controlled by RNA splicing factors CUG-BP and hnRNPM also correlated with disrupted cell barrier, compromised angiogenesis, and enhanced monocyte adhesion [49]. During the progression of diabetic retinopathy, downregulation of miR-200b results in the elevation of its target VEGF mRNA and protein, resulting in increased vascular permeability and angiogenesis [61]. QKI increases STAT3 mRNA stability by binding to its 3’ untranslated region, and upregulated expression of STAT3 promotes Pulmonary artery smooth muscle cell (PASMC) proliferation in vitro and in vivo. In another instance, QKI expression regulation by miR-214 proved to be highly effective against compensatory arteriogenesis and angiogenesis. miR-214 directly targets Quaking, resulting in reduced pro-angiogenic growth factor expression and inhibited EC sprouting [62]. Studies by Justice [52 and van Mil [62] furthermore shows, the roles of QKI in acute coronary syndrome (ACS). It has been shown that HCG11, which is regulated by QKI5 through a feedback mechanism acts as a sponge for miR-26b-5p. Further, QKI5 was speculated as the target of miR-26b-5p during ACS [63]. QKI-6 was found to induce VSMC differentiation from induced pluripotent stem cells (iPSCs), showing the role of QKI in diabetes pathology [33]. During ischemia, PDGF-BB stimulation induced QKI-6, promoting HDAC7 splicing, activation of SM22, and iPS-VSMC differentiation, thus demonstrating QKI-6 is critical for the modulation of HDAC7 splicing for regulating iPS-VSMCs differentiation and functionality [33, 64]. These studies indicate that QKI, as an RBP, plays significant role in EC pathology, especially in diabetes and cardiovascular disorders.

Tumor angiogenesis and metastasis are major contributors to the mortality associated with cancers. Several reports have demonstrated direct roles of QKI in tumor EC biology, as well as how cancer cell expression of QKI regulates tumor angiogenesis. Prior work in lung cancer demonstrated that tumor ECs have reduced miR-200 family expression, resulting in increased expression of all QKI isoforms. QKI stabilization of CCND1 in tumor ECs in turn promoted tumor EC proliferation, tumor angiogenesis and metastases [36]. The binding of QKI-5 at the 3’UTR of signal transducer and activator of transcription 3 (STAT3) promotes stabilization and phosphorylation, thus vascular endothelial growth factor receptor 2 (VEGFR2) and VE-cadherin could be activated and stabilized, resulting in increased neovascularization and angiogenesis [50]. In renal cell carcinoma (RCC), CircSPIRE1 was identified as a metastasis-inhibiting circular RNA that suppresses the mesenchymal state through upregulating QKI expression. Positive feedback by QKI enhanced circSPIRE1 expression, its packaging and delivery into ECs via exosomes, thus resulting in reduced tumor-associated angiogenesis in RCC [65]. Interestingly, in aggressive glioblastoma, NF-κB induced miR-148a expression, resulting in suppression of its target gene, QKI. Because QKI acts as a negative regulator of TGF-β, the reduced QKI resulted in enhanced TGF-β/Smad signaling and tumor angiogenesis [66].

## Future mechanistic insights and conclusion

Due to its impact on the physiology, function, and pathology of endothelial cells, understanding the regulation of QKI isoform expression and localization is of the utmost importance. There is limited knowledge regarding the regulation of different isoform expression of QKI, including how the isoforms auto-regulate themselves, and each other. For instance, an increase in QKI transcription levels triggers a shift to favor the expression of QKI-6 and QKI-7, which is regulated by QKI-5 [2, 10]. QKI-5, with its nuclear localization signal, impacts the location of other isoforms, increasing the nuclear localization of QKI-6 and QKI-7 [5]. Recently, several other unique functions of the QKI isoforms have emerged. For example, efforts to study RBP distribution patterns in the nucleus and screening for chromatin-enriched RBPs (Che-RBPs) revealed that QKI-5 is a direct transcriptional activator during monocytic differentiation. QKI-5 was found to activate transcription of several critical monocytic differentiation-associated genes, notably CXCL2 [30]. Using a genetic mouse model of *Qki* loss, QKI was found to enhance cholesterol biosynthesis by recruiting Srebp2 and Pol II in the promoter regions of cholesterol biosynthesis genes. This observation demonstrated that in addition to QKI’s function as a transcription co-activator, QKI can also directly interact with single-stranded DNA to maintaining cholesterol levels [40].

In this review, we summarize some of the emerging roles of QKI in post-transcriptional modifications and regulations in EC biology and pathophysiology. During physiologic and pathologic EC conditions, QKI regulates alternative splicing, polyadenylation, mRNA stabilization, mRNA subcellular location, and non-coding RNAs, which are largely regulated by their binding to the QRE elements of targeted pre-mRNA or mRNA. Consequently, largely due to the roles in RNA splicing and mRNA stability, QKI isoforms have important contributions to the regulation of EC proliferation, differentiation, and tissue-specific lineage assignment, thus nominating QKI as a potential target for angiogenesis-based therapies.

## Data Availability

No datasets were generated or analysed during the current study.

## References

[1] Mehta M et al (2022) RNA binding proteins (RBPs) and their role in DNA damage and radiation response in cancer. Adv Drug Deliv Rev 191:114569 36252617 10.1016/j.addr.2022.114569 PMC10411638

[2] Zhu W et al (2025) RNA-binding protein quaking: a multifunctional regulator in tumour progression. Ann Med 57(1):2443046 39711373 10.1080/07853890.2024.2443046 PMC11703483

[3] Teplova M et al (2013) Structure-function studies of STAR family Quaking proteins bound to their in vivo RNA target sites. Genes Dev 27(8):928–940 23630077 10.1101/gad.216531.113 PMC3650229

[4] Galarneau A, Richard S (2009) The STAR RNA binding proteins GLD-1, QKI, SAM68 and SLM-2 bind bipartite RNA motifs. BMC Mol Biol 10:47 19457263 10.1186/1471-2199-10-47 PMC2697983

[5] Neumann DP, Goodall GJ, Gregory PA (2022) The quaking RNA-binding proteins as regulators of cell differentiation. Wiley Interdiscip Rev RNA 13(6):e1724 35298877 10.1002/wrna.1724 PMC9786888

[6] Vernet C, Artzt K (1997) STAR, a gene family involved in signal transduction and activation of RNA. Trends Genet 13(12):479–484 9433137 10.1016/s0168-9525(97)01269-9

[7] Bohnsack BL et al (2006) Visceral endoderm function is regulated by quaking and required for vascular development. Genesis 44(2):93–104 16470614 10.1002/gene.20189

[8] Chatterji P, Rustgi AK (2018) RNA binding proteins in intestinal epithelial biology and colorectal cancer. Trends Mol Med 24(5):490–506 29627433 10.1016/j.molmed.2018.03.008 PMC5927824

[9] Hayakawa-Yano Y et al (2017) An RNA-binding protein, Qki5, regulates embryonic neural stem cells through pre-mRNA processing in cell adhesion signaling. Genes Dev 31(18):1910–1925 29021239 10.1101/gad.300822.117 PMC5693031

[10] Fagg WS et al (2017) Autogenous cross-regulation of Quaking mRNA processing and translation balances Quaking functions in splicing and translation. Genes Dev 31(18):1894–1909 29021242 10.1101/gad.302059.117 PMC5695090

[11] Chen X et al (2021) QKI is a critical pre-mRNA alternative splicing regulator of cardiac myofibrillogenesis and contractile function. Nat Commun 12(1):89 33397958 10.1038/s41467-020-20327-5 PMC7782589

[12] Zhang R et al (2021) Quaking I-5 protein inhibits invasion and migration of kidney renal clear cell carcinoma via inhibiting epithelial-mesenchymal transition suppression through the regulation of microRNA 200c. Transl Androl Urol 10(10):3800–3814 34804823 10.21037/tau-21-833 PMC8575590

[13] Pillman KA et al (2018) Mir-200/375 control epithelial plasticity-associated alternative splicing by repressing the RNA-binding protein Quaking. EMBO J. 10.15252/embj.201899016 29871889 10.15252/embj.201899016 PMC6028027

[14] Kondo T et al (1999) Genomic organization and expression analysis of the mouse qkI locus. Mamm Genome 10(7):662–669 10384037 10.1007/s003359901068

[15] Chen T et al (1997) Self-association of the single-KH-domain family members Sam68, GRP33, GLD-1, and Qk1: role of the KH domain. Mol Cell Biol 17(10):5707–5718 9315629 10.1128/mcb.17.10.5707 PMC232419

[16] Chen T, Richard S (1998) Structure-function analysis of Qk1: a lethal point mutation in mouse quaking prevents homodimerization. Mol Cell Biol 18(8):4863–4871 9671495 10.1128/mcb.18.8.4863 PMC109071

[17] Zorn AM, Krieg PA (1997) The KH domain protein encoded by quaking functions as a dimer and is essential for notochord development in Xenopus embryos. Genes Dev 11(17):2176–2190 9303534 10.1101/gad.11.17.2176 PMC275400

[18] Hasan MK, Brady LJ (2024) Nucleic acid-binding KH domain proteins influence a spectrum of biological pathways including as part of membrane-localized complexes. J Struct Biol X 10:100106 39040530 10.1016/j.yjsbx.2024.100106 PMC11261784

[19] Lu J, Gao FH (2016) Role and molecular mechanism of heterogeneous nuclear ribonucleoprotein K in tumor development and progression. Biomed Rep 4(6):657–663 27284403 10.3892/br.2016.642 PMC4887935

[20] Gallardo M et al (2016) Aberrant hnRNP K expression: all roads lead to cancer. Cell Cycle 15(12):1552–1557 27049467 10.1080/15384101.2016.1164372 PMC4934053

[21] Sidiqi M et al (2005) Structure and RNA binding of the third KH domain of poly(C)-binding protein 1. Nucleic Acids Res 33(4):1213–1221 15731341 10.1093/nar/gki265 PMC549569

[22] Yadav M et al (2021) The KH domain facilitates the substrate specificity and unwinding processivity of DDX43 helicase. J Biol Chem 296:100085 33199368 10.1074/jbc.RA120.015824 PMC7949032

[23] Hollingworth D et al (2012) KH domains with impaired nucleic acid binding as a tool for functional analysis. Nucleic Acids Res 40(14):6873–6886 22547390 10.1093/nar/gks368 PMC3413153

[24] Beuck C et al (2012) Structural analysis of the quaking homodimerization interface. J Mol Biol 423(5):766–781 22982292 10.1016/j.jmb.2012.08.027 PMC3472039

[25] Hong D, Jeong S (2023) 3’UTR diversity: expanding repertoire of RNA alterations in human mRNAs. Mol Cells 46(1):48–56 36697237 10.14348/molcells.2023.0003 PMC9880603

[26] Darbelli L et al (2017) Transcriptome profiling of mouse brains with qkI-deficient oligodendrocytes reveals major alternative splicing defects including self-splicing. Sci Rep 7(1):7554 28790308 10.1038/s41598-017-06211-1 PMC5548867

[27] Chen Y et al (2007) The selective RNA-binding protein quaking I (QKI) is necessary and sufficient for promoting oligodendroglia differentiation. J Biol Chem 282(32):23553–23560 17575274 10.1074/jbc.M702045200

[28] Wu JI et al (2002) Function of quaking in myelination: regulation of alternative splicing. Proc Natl Acad Sci U S A 99(7):4233–4238 11917126 10.1073/pnas.072090399 PMC123631

[29] Wu J et al (1999) The quaking I-5 protein (QKI-5) has a novel nuclear localization signal and shuttles between the nucleus and the cytoplasm. J Biol Chem 274(41):29202–29210 10506177 10.1074/jbc.274.41.29202

[30] Ren Y et al (2021) A global screening identifies chromatin-enriched RNA-binding proteins and the transcriptional regulatory activity of QKI5 during monocytic differentiation. Genome Biol 22(1):290 34649616 10.1186/s13059-021-02508-7 PMC8518180

[31] Hardy RJ et al (1996) Neural cell type-specific expression of QKI proteins is altered in quakingviable mutant mice. J Neurosci 16(24):7941–7949 8987822 10.1523/JNEUROSCI.16-24-07941.1996 PMC6579212

[32] Wang Y et al (2013) The QKI-5 and QKI-6 RNA binding proteins regulate the expression of microRNA 7 in glial cells. Mol Cell Biol 33(6):1233–1243 23319046 10.1128/MCB.01604-12 PMC3592017

[33] Caines R et al (2019) The RNA-binding protein QKI controls alternative splicing in vascular cells, producing an effective model for therapy. J Cell Sci. 10.1242/jcs.230276 31331967 10.1242/jcs.230276

[34] Wu Y et al (2017) Microrna-214 regulates smooth muscle cell differentiation from stem cells by targeting RNA-binding protein QKI. Oncotarget 8(12):19866–19878 28186995 10.18632/oncotarget.15189 PMC5386729

[35] Shu P et al (2017) Microrna-214 modulates neural progenitor cell differentiation by targeting Quaking during cerebral cortex development. Sci Rep 7(1):8014 28808337 10.1038/s41598-017-08450-8 PMC5556025

[36] Azam SH et al (2019) Quaking orchestrates a post-transcriptional regulatory network of endothelial cell cycle progression critical to angiogenesis and metastasis. Oncogene 38(26):5191–5210 30918328 10.1038/s41388-019-0786-6 PMC6597267

[37] Mukohyama J et al (2019) Mir-221 targets QKI to enhance the tumorigenic capacity of human colorectal cancer stem cells. Cancer Res 79(20):5151–5158 31416845 10.1158/0008-5472.CAN-18-3544 PMC6801097

[38] Zhao Z et al (2023) QKI shuttles internal m(7)G-modified transcripts into stress granules and modulates mRNA metabolism. Cell 186(15):3208-3226 e27 37379838 10.1016/j.cell.2023.05.047 PMC10527483

[39] Pedersen SK et al (2014) CAHM, a long non-coding RNA gene hypermethylated in colorectal neoplasia. Epigenetics 9(8):1071–1082 24799664 10.4161/epi.29046 PMC4164492

[40] Shin S et al (2021) Qki activates Srebp2-mediated cholesterol biosynthesis for maintenance of eye lens transparency. Nat Commun 12(1):3005 34021134 10.1038/s41467-021-22782-0 PMC8139980

[41] Manna PR et al (2023) Expression and function of StAR in cancerous and non-cancerous human and mouse breast tissues: new insights into diagnosis and treatment of hormone-sensitive breast cancer. Int J Mol Sci. 10.3390/ijms24010758 36614200 10.3390/ijms24010758 PMC9820903

[42] Feracci M et al (2016) Structural basis of RNA recognition and dimerization by the STAR proteins T-STAR and Sam68. Nat Commun 7:10355 26758068 10.1038/ncomms10355 PMC4735526

[43] Tao Y et al (2024) Alternative splicing and related RNA binding proteins in human health and disease. Signal Transduct Target Ther 9(1):26 38302461 10.1038/s41392-024-01734-2 PMC10835012

[44] Guo Z et al (2024) The multifaceted role of quaking protein in neuropsychiatric disorders and tumor progression. Front Neurosci 18:1341114 39479357 10.3389/fnins.2024.1341114 PMC11521838

[45] Zhang Y et al (2003) Tyrosine phosphorylation of QKI mediates developmental signals to regulate mRNA metabolism. EMBO J 22(8):1801–1810 12682013 10.1093/emboj/cdg171 PMC154463

[46] Paronetto MP et al (2007) The RNA-binding protein Sam68 modulates the alternative splicing of Bcl-x. J Cell Biol 176(7):929–939 17371836 10.1083/jcb.200701005 PMC2064079

[47] Larocque D et al (2009) The QKI-6 and QKI-7 RNA binding proteins block proliferation and promote Schwann cell myelination. PLoS ONE 4(6):e5867 19517016 10.1371/journal.pone.0005867 PMC2690695

[48] de Bruin RG et al (2016) The RNA-binding protein quaking maintains endothelial barrier function and affects VE-cadherin and beta-catenin protein expression. Sci Rep 6:21643 26905650 10.1038/srep21643 PMC4764852

[49] Yang C et al (2020) Targeting QKI-7 in vivo restores endothelial cell function in diabetes. Nat Commun 11(1):3812 32732889 10.1038/s41467-020-17468-y PMC7393072

[50] Cochrane A et al (2017) Quaking is a key regulator of endothelial cell differentiation, neovascularization, and angiogenesis. Stem Cells 35(4):952–966 28207177 10.1002/stem.2594 PMC5396345

[51] Huq MD et al (2006) Regulation of retinal dehydrogenases and retinoic acid synthesis by cholesterol metabolites. EMBO J 25(13):3203–3213 16763553 10.1038/sj.emboj.7601181 PMC1500992

[52] Justice MJ, Hirschi KK (2010) The role of quaking in mammalian embryonic development. Adv Exp Med Biol 693:82–92 21189687 10.1007/978-1-4419-7005-3_6

[53] Papaioannou VE, Behringer RR (2012) Early embryonic lethality in genetically engineered mice: diagnosis and phenotypic analysis. Vet Pathol 49(1):64–70 21233329 10.1177/0300985810395725 PMC3134574

[54] Li Z et al (2003) Defective smooth muscle development in qkI-deficient mice. Dev Growth Differ 45(5–6):449–462 14706070 10.1111/j.1440-169x.2003.00712.x

[55] van der Veer EP et al (2013) Quaking, an RNA-binding protein, is a critical regulator of vascular smooth muscle cell phenotype. Circ Res 113(9):1065–1075 23963726 10.1161/CIRCRESAHA.113.301302

[56] Cao G et al (2022) How vascular smooth muscle cell phenotype switching contributes to vascular disease. Cell Commun Signal 20(1):180 36411459 10.1186/s12964-022-00993-2 PMC9677683

[57] Yang C et al (2018) RBPs play important roles in vascular endothelial dysfunction under diabetic conditions. Front Physiol 9:1310 30294283 10.3389/fphys.2018.01310 PMC6158626

[58] Montanes-Agudo P et al (2023) The RNA-binding protein QKI governs a muscle-specific alternative splicing program that shapes the contractile function of cardiomyocytes. Cardiovasc Res 119(5):1161–1174 36627242 10.1093/cvr/cvad007 PMC10202634

[59] Woodcock CC et al (2021) Matrix stiffening induces a pathogenic QKI-miR-7-SRSF1 signaling axis in pulmonary arterial endothelial cells. Am J Physiol Lung Cell Mol Physiol 320(5):L726–L738 33565360 10.1152/ajplung.00407.2020 PMC8174827

[60] Yang DR et al (2024) Endothelial dysfunction in vascular complications of diabetes: a comprehensive review of mechanisms and implications. Front Endocrinol (Lausanne) 15:1359255 38645427 10.3389/fendo.2024.1359255 PMC11026568

[61] McArthur K et al (2011) MicroRNA-200b regulates vascular endothelial growth factor-mediated alterations in diabetic retinopathy. Diabetes 60(4):1314–1323 21357793 10.2337/db10-1557 PMC3064105

[62] van Mil A et al (2012) Microrna-214 inhibits angiogenesis by targeting Quaking and reducing angiogenic growth factor release. Cardiovasc Res 93(4):655–665 22227154 10.1093/cvr/cvs003

[63] Du J et al (2020) LncRNA HCG11/miR-26b-5p/QKI5 feedback loop reversed high glucose-induced proliferation and angiogenesis inhibition of HUVECs. J Cell Mol Med 24(24):14231–14246 33128346 10.1111/jcmm.16040 PMC7753996

[64] Zhou B et al (2011) Splicing of histone deacetylase 7 modulates smooth muscle cell proliferation and neointima formation through nuclear beta-catenin translocation. Arterioscler Thromb Vasc Biol 31(11):2676–2684 21836063 10.1161/ATVBAHA.111.230888

[65] Shu G et al (2023) Exosomal circSPIRE1 mediates glycosylation of E-cadherin to suppress metastasis of renal cell carcinoma. Oncogene 42(22):1802–1820 37046045 10.1038/s41388-023-02678-7 PMC10238271

[66] Wang H et al (2015) NF-kappaB induces miR-148a to sustain TGF-beta/Smad signaling activation in glioblastoma. Mol Cancer 14:2 25971746 10.1186/1476-4598-14-2 PMC4429406

